# Emotional State Transitions in Trauma-Exposed Individuals With and Without Posttraumatic Stress Disorder

**DOI:** 10.1001/jamanetworkopen.2024.6813

**Published:** 2024-04-16

**Authors:** Nachshon Korem, Or Duek, Tobias Spiller, Ziv Ben-Zion, Ifat Levy, Ilan Harpaz-Rotem

**Affiliations:** 1 Department of Psychiatry, Yale University School of Medicine, New Haven, Connecticut; 2 Department of Comparative Medicine, Yale University School of Medicine, New Haven, Connecticut; 3 US Department of Veterans Affairs (VA) National Center for Posttraumatic Stress Disorder, VA Connecticut Healthcare System, West Haven; 4 Department of Epidemiology, Biostatistics, and Community Health Sciences, Ben-Gurion University of the Negev, Beer-Sheva, Israel; 5 Faculty of Medicine, University of Zurich, Zurich, Switzerland; 6 Department of Consultation-Liaison Psychiatry and Psychosomatic Medicine, University Hospital Zurich, Zurich, Switzerland; 7 Department of Psychology, Yale University, New Haven, Connecticut; 8 Department of Neuroscience, Yale University, New Haven, Connecticut; 9 Wu Tsai Institute, Yale University New Haven, New Haven, Connecticut

## Abstract

**Importance:**

Posttraumatic stress disorder (PTSD) is marked by the contrasting symptoms of hyperemotional reactivity and emotional numbing (ie, reduced emotional reactivity). Comprehending the mechanism that governs the transition between neutral and negative emotional states is crucial for developing targeted therapeutic strategies.

**Objectives:**

To explore whether individuals with PTSD experience a more pronounced shift between neutral and negative emotional states and how the intensity of emotional numbing symptoms impacts this shift.

**Design, Setting, and Participants:**

This cross-sectional study used hierarchical bayesian modeling to fit a 5-parameter logistic regression to analyze the valence ratings of images. The aim was to compare the curve’s slope between groups and explore its association with the severity of emotional numbing symptoms. The study was conducted online, using 35 images with a valence range from highly negative to neutral. The rating of these images was used to assess the emotional responses of the participants. The study recruited trauma-exposed individuals (witnessed or experienced life-threatening incident, violent assault, or someone being killed) between January 17 and March 8, 2023. Participants completed the PTSD Checklist for the *Diagnostic and Statistical Manual of Mental Disorders* (Fifth Edition) (*DSM-5*) (PCL-5).

**Exposure:**

On the basis of *DSM-5* criteria (endorsing at least 1 symptom from clusters B and C and 2 from D and E), participants were categorized as having probable PTSD (pPTSD) or as trauma-exposed controls (TECs).

**Main Outcomes and Measures:**

The main outcome was the slope parameter (b) of the logistic curve fitted to the valence rating. The slope parameter indicates the rate at which emotional response intensity changes with stimulus valence, reflecting how quickly the transition occurs between neutral and negatively valenced states. The secondary outcome was the association between emotional numbing (PCL-5 items 12-14) and the slope parameter.

**Results:**

A total of 1440 trauma-exposed individuals were included. The pPTSD group (n = 445) was younger (mean [SD] age, 36.1 [10.9] years) compared with the TEC group (mean [SD] age, 41.5 [13.3] years; *P* < .001). Sex distribution (427 women in the TEC group vs 230 in the pPTSD group) did not significantly differ between groups (*P* = .67). The pPTSD group exhibited a steeper slope (mean slope difference, −0.255; 89% highest posterior density [HPD], −0.340 to −0.171) compared with the controls. Across all individuals (n = 1440), a robust association was found between the slope and emotional numbing severity (mean [SD] additive value, 0.100 [0.031]; 89% HPD, 0.051-0.15). Additional analysis controlling for age confirmed the association between emotional numbing and transition sharpness (mean [SD] additive value, 0.108 [0.032]; 89% HPD, 0.056-0.159), without evidence of an age-related association (mean [SD] additive value, 0.031 [0.033]; 89% HPD, −0.022 to 0.083).

**Conclusions and Relevance:**

These findings support that individuals with PTSD undergo rapid transitions between neutral and negative emotional states, a phenomenon intensified by the severity of emotional numbing symptoms. Therapeutic interventions aimed at moderating these swift emotional transitions could potentially alleviate PTSD symptoms.

## Key Points

**Question:**

How does the transition between neutral and negative emotional states differ between trauma-exposed individuals with and without posttraumatic stress disorder (PTSD)?

**Findings:**

In this cross-sectional study of 1440 trauma-exposed individuals, participants who met the criteria for PTSD exhibited a significantly faster transition rate between neutral and negative emotional states than controls. A higher transition rate was further associated with increased symptoms of emotional numbing.

**Meaning:**

This study suggests that rapid shifts between neutral and negative emotional states in PTSD may bridge these seemingly contrasting symptoms, offering new insights for therapeutic strategies.

## Teaser

This cross-sectional study examines whether trauma-exposed individuals with posttraumatic stress disorder have a sharper transition between neutral and negative emotional states compared with trauma-exposed individuals without posttraumatic stress disorder.

## Introduction

**Figure 1.**
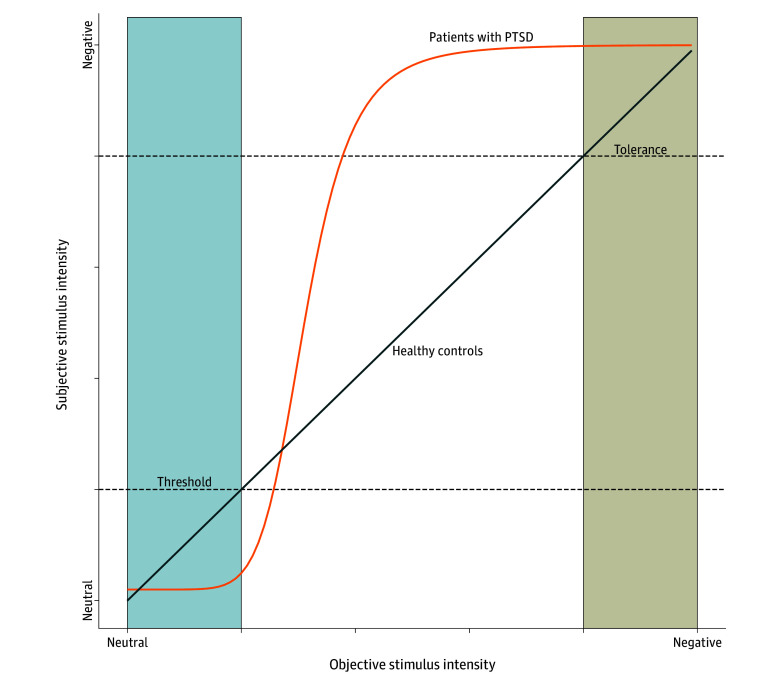
Illustration of a Theoretical Valenced Response of Patients With Posttraumatic Stress Disorder (PTSD) In the PTSD group, the transition between emotional responses happens more rapidly. Boxes represent the area where healthy participants consider a scenario as mildly valenced (blue) or highly valenced (beige). During mildly valenced situations, people diagnosed with PTSD do not respond; they go into a numb state—a high threshold. However, they reach their maximum capacity in response to less severe stimuli—low tolerance.

The nature of emotional processing in posttraumatic stress disorder (PTSD) is a matter of much debate. One symptom central to this debate is emotional numbing—a restricted capacity to experience positive emotions.^1^ However, emotional numbing has been suggested to affect negative emotions as well.^2,3,4^ In contrast, most other symptoms of PTSD are linked to hyperreactivity to emotional stimuli.^5^ Although it is still a matter of contention whether the exaggerated response is general or specific to trauma-related stimuli,^6^ there is overwhelming evidence for an amplified response in self-reports,^7^ physiologic measures,^8,9^ and neural activation.^10,11,12^ A potential resolution to the hypo-hyper emotionality paradox is that PTSD may be associated with a faster transition from no emotional response (numb state) to hyperreactivity, closer to an all-or-nothing response. Mathematically, we can visualize the emotional response as a logistic curve instead of a linear curve (Figure 1).

In contrast to the focus on highly valenced emotional stimuli used in PTSD research (eg, trauma scripts^13^), studies on pain in PTSD often use low-mild stimuli.^14,15^ Because pain and emotions are closely interconnected,^16^ we can look at the response to low-mild pain to learn about the emotional response. One prominent feature of PTSD is an increase in the pain threshold, particularly after exposure to stress, known as stress-induced analgesia.^17,18^ Individuals with PTSD, however, also exhibit lower pain tolerance, which means they become overwhelmed by pain more quickly.^19,20^ A recent study^14^ found that veterans with PTSD had a lower amygdala activation to mild pain compared with trauma-exposed veterans without PTSD. Furthermore, amygdala activation to mild pain was negatively associated with the severity of emotional numbing symptoms. On the other hand, no effects were found in the insula, a core region of the pain matrix.^21^ These results suggest that the numbing experienced in PTSD is closely linked to the psychological and emotional processing of pain beyond the physiologic aspects alone. Consequently, this finding supports the idea that the response to emotional stimuli in PTSD may share a similar pattern of high threshold, low tolerance; however, to our knowledge, this idea has never been tested before.

One common approach to studying emotions in PTSD is through valence images.^7^ These images are derived from valenced image databases, providing stimuli with predefined valence and arousal norms.^22^ We can identify deviations associated with specific groups by comparing psychopathologic conditions to these norms. In a previous study, people diagnosed with PTSD differed from trauma-exposed healthy controls only in their ratings of trauma-related images.^7^ However, this comparison grouped images into negative, trauma-related negative, and neutral categories, masking the transition phase between neutral and negative emotions. Transitioning from discrete comparisons of highly negative and neutral images to a continuous negative-to-neutral scale together with computational tools offers a solution because it creates a function that describes the dynamics of people’s valence reaction (ie, the curvature). One way to estimate the high-threshold, low-tolerance response is to transition from estimating emotional response as a linear curve to estimating it as a logistic curve. The logistic curve can be described using several parameters, one being the slope, which determines how quickly the transition occurs between the neutral and negative states. If PTSD, and more specifically the emotional numbing cluster, as defined by the 8-factor model of PTSD,^23^ is indeed associated with a steeper slope, it would represent a significant step toward reconciling the hypo-hyper emotionality paradox in PTSD.

In this study, we developed a computational approach to investigate the transition between neutral and valence states in individuals diagnosed with PTSD. Drawing on our hypothesis (Figure 1), we investigate whether PTSD is characterized by a notably sharper transition (a higher slope) between neutral and negative emotional states compared with trauma-exposed individuals who did not develop the disorder. Moreover, we sought to explore whether the transition is positively associated with the severity of emotional numbing symptoms experienced by the individuals.

## Methods

### Participants

Using an online platform (Prolific), we recruited 1463 trauma-exposed (witnessing or experiencing a car crash, a violent act, or someone being killed) participants for this cross-sectional study. All participants completed the PTSD Checklist for *DSM-5* (PCL-5).^24^ Participants received a diagnosis of probable PTSD (pPTSD) if they had a PCL-5 score of more than 33^25^ and endorsed (rated as ≥2) at least 1 intrusion symptom (items 1-5), 1 avoidance symptom (items 6-7), 2 negative alterations in cognitions and mood symptoms (items 8-14), and 2 alterations in arousal and reactivity symptoms, similar to the requirement pose by the *Diagnostic and Statistical Manual of Mental Disorders* (Fifth Edition) (*DSM-5*).^26^ The rest of the participants were categorized as trauma-exposed controls (TECs). Self-reported race data were collected for demographic information. The study was approved by the institutional review board of Yale University, and written informed consent was obtained from all participants. Additionally, participants were provided with the telephone number and email of a trained clinician to contact in case they felt triggered or overwhelmed by the content of the study. This report followed the Strengthening the Reporting of Observational Studies in Epidemiology (STROBE) reporting guideline.

### Emotional Numbing

Emotional numbing symptoms were assessed using items 12 to 14 of the PCL-5: “Loss of interest in activities that you used to enjoy?”; “Feeling distant or cut off from other people?”; and “Trouble experiencing positive feelings (for example, being unable to feel happiness or have loving feelings for people close to you)?” (possible scores, 0-12).^25^ These scores were based on the 8-factor model of PTSD.^14,23^

### Stimuli

The stimuli comprised 40 images from the Nencki Affective Picture System (NAPS).^22^ We chose images of faces, people, and animals from the data set. We sorted the images based on the mean valence starting from the most negative; we chose images with a gradual increase in mean valence. On the basis of NAPS standards, 35 images had a valence rating ranging from the most negative (1.33) to neutral (5), and 5 images had positive valence ratings (5.63-7.09); for a list of the names of the images, see eTable 1 in Supplement 1. The images selected spanned various categories to minimize the likelihood of triggering specific trauma. The positive images were used to reduce negativity bias and were omitted from the analysis.^27^

### Procedure

Participants were recruited online (Prolific) between January 17 and March 8, 2023. Two to 5 days before participating in the experiment, all participants underwent a prescreening session involving questionnaires to ensure they met the criteria for trauma exposure (criterion A) using the Life Events Checklist for *DSM-5*.^28^ Prescreening sessions included several questions about their lifestyle (eg, “Are you a vegetarian?”; “Do you prefer public transportation or private car?”; “Do you prefer cats or dogs?”) to disguise the main interest of the screening and prevent people from overreporting witnessing or experiencing trauma in the hope of participating in the full study. During the experiment, participants used the Qualtrics platform (Qualtrics XM) to complete the PCL-5 before proceeding to the image rating task. The images were displayed one by one in random order; each image remained on the screen until participants pressed the advance button. Participants used a visual analog scale (VAS) to rate the images from unpleasant to pleasant. They had to move the scale to advance to the next image. Attention checks were used to ensure participants paid attention. Participants whose ratings deviated from the NAPS values by more than 3 SDs on more than 2 occasions were identified as outliers and were excluded from further analysis (n = 83).

### Statistical Analysis

Given the hierarchical structure of our data, we opted for hierarchical bayesian modeling (HBM). This approach provides access to the full posterior distribution for more nuanced inferences and accommodates the inclusion of prior information to enhance parameter estimates. Consequently, we fitted a 5-parameter logistic (5-PL) regression model using HBM separately to the pPTSD and TEC data using the following equation (eFigure in Supplement 1):

f(x) = d+(a-d)/[1+(x/c)^b^]^g^

a ~ β(α_1_, α_2_)

b ~ Normal(μ_b_**_,_** 1, 0.5, ∞)

c ~ Normal(μ_c_**_,_** 1, 0.5, 5)

d ~ Normal(μ_d_**_,_** 1, 0.5, ∞)

g ~ Normal(μ_g_**_,_** 1, 0.8, ∞)

α_1_ ~ Normal(2**_,_** 1, 1, ∞)

α_2_ ~ Normal(10**_,_** 1, -∞, ∞)

μ_b_ ~ Normal(2**_,_** 1, 1, ∞)

μ_c_ ~ Normal(2**_,_** 1, 0.5, ∞)

μ_d_ ~ Normal(2**_,_** 1, 0, ∞)

μ_g_ ~ Normal(4**_,_** 1, 1, ∞),

where a represents the minimum asymptote, corresponding to the ratings of the most negative images; b refers to the Hill slope, indicating the curve’s steepness; c is the inflection point, where participants rated the images as having a medium valence, approximately where y ~ (d − a)/2; d represents the maximum asymptote, reflecting the ratings given to neutral images (neutral); and g represents the asymmetry factor, with a value of 1 indicating a symmetrical curve around c. Prior selection was done based on the data properties and simulations. The data from the NAPS were on a scale of 1 to 9, whereas participants’ ratings on the VAS were 0 to 1. Hence, we expected the responses to the most negative images on the VAS to be close to 0 and the response to the neutral close to 0.5. Because our image selection method aimed to get images with a gradual increase and should approach linear in healthy individuals, we used priors that were able to also converge on a linear slope. For the a parameter, we used a β distribution with a mean close to 0. The rest of the parameters used truncated normal distribution because negative values were impossible (all ratings were positive; ie, 0-1). Through simulations, we refined parameters to enhance convergence, minimize rHat, and optimize sampling. A hierarchical approach was adopted for parameter modeling, ensuring sensitivity to population-level variations.

This model was compared to a linear model with the following equation:

f(x) = bx + a

a ~ Normal(0**_,_** 0.2, 0, ∞)

b ~ Normal(0**_,_** 0.5, 0, ∞).

We extracted the posterior distribution of each parameter and subtracted the pPTSD distribution from the TEC posterior distribution. Robust differences were considered if 0 fell outside the 89% highest posterior density (HPD).^14,29,30^ To assess the association between emotional numbing and the slope (b) parameter, we adjusted the slope parameter to be the result of a linear regression with participant-specific b as the intercept and a common slope (EN). That is, we used a fixed slope random intercept approach where B replaces the slope (b) in the 5-PL regression equation. Emotional numbing was *z*-transformed to improve model convergence. The equation is as follows:

B_i_ = Emotional Numbing *z* score × EN + b_i_

EN ~ Normal(0**_,_** 1, -∞, ∞).

A robust association was considered if the 89% HPD of the slope did not include 0. All models converge with an rHat less than 1.01 and effective sampling rate greater than 1000. All analyses were conducted in Python, version 3.9.13 (Python Software Foundation, using the PyMC (version 4.1.7)^31^ and ArviZ (version 0.12.1)^32^ packages. We used the No-U-Turn Sampler for Markov Chain Monte Carlo inference, adhering to PyMC’s default settings: 1000 draws, 1000 tuning steps, and an 80% acceptance rate, without thinning.

### Model Comparison

We used a leave-one-out (LOO) cross-validation method to compare the models by estimating out-of-sample predictive fit.^33^ This method partitions the data into train and test data and fits the training-based data on the holdout test data, evaluating the fit. Unlike log likelihood, LOO measures expected log pointwise predictive density.^34^ Thus, a higher LOO indicates a better fit. All code is available on GitHub.^35^

## Results

### Sample Description and Demographics

A total of 1440 trauma-exposed individuals (mean [SD] age, 39.67 [12.8] years; 723 [52.4%] male and 657 [47.6%] female; 106 [7.4%] African American or Black, 9 [0.6%] American Indian or Alaska Native, 66 [4.6%] Asian, 1087 [75.3%] White, 79 [5.5%] multiracial, 23 [1.6%] other (race not specified), and 10 [0.7%] preferred not to say) were studied. The demographic and clinical characteristics of the sample are presented in Table 1. We found a significant age difference between the pPTSD and TEC groups (mean [SD] age, 36.1 [10.9] years for the pPTSD group compared with 41.5 [13.3] years for the TEC group; *P* < .001). Sex distribution (427 women in the TEC group vs 230 in the pPTSD group) did not significantly differ between groups (*P* = .67).

**Table 1.** Demographic and Clinical Characteristics of the Sample^a^

Characteristic	Trauma exposed controls (n = 925)	Patients with probable PTSD (n = 455)	*P* value
Age, mean (SD), y	41.5 (13.3)	36.1 (10.9)	<.001
Sex			
Male	498 (53.8)	225 (49.4)	.67
Female	427 (46.2)	230 (50.6)	
PTSD checklist for *DSM-5* score, mean (SD) [range)	17.81 (10.14) [0-56]	46.52 (9.72) [33-76]	<.001
Emotional numbing score (items 12-14), mean (SD) [range]	2.95 (2.63) [0-12]	7.94 (2.49) [0-12]	<.001
Educational level			
Some high school or less	12 (1.3)	5 (1.1)	<.001
High school diploma or GED	125 (13.5)	85 (18.7)	
Some college but no degree	189 (20.4)	133 (29.2)	
Associate’s or technical degree	120 (13.0)	47 (10.3)	
Bachelor’s degree	322 (34.8)	141 (31.0)	
Graduate or professional degree	155 (16.8)	44 (9.7)	
Prefer not to say	2 (0.2)	0	
Ethnicity			
Spanish, Hispanic, or Latino	90 (9.7)	46 (10.3)	.90
Not Spanish, Hispanic, or Latino	835 (90.3)	409 (90.0)	
Race^b^			
African American or Black	62 (6.7)	44 (9.7)	.23
American Indian or Alaska Native	5 (0.5)	4 (0.9)	
Asian	41 (4.4)	25 (5.5)	
White	741 (80.1)	343 (75.4)	
Multiracial	53 (5.7)	26 (5.7)	
Other^c^	14 (1.5)	9 (2.0)	
Prefer not to say	9 (1.0)	1 (0.2)	
Marital status			
Married	345 (37.3)	135 (29.7)	.04
Living with a partner	145 (15.7)	83 (18.2)	
Widowed	14 (1.5)	6 (1.3)	
Divorced or separated	100 (10.8)	44 (9.7)	
Never been married	321 (34.7)	187 (41.1)	

Abbreviations: *DSM-5*, *Diagnostic and Statistical Manual of Mental Disorders* (Fifth Edition); GED, General Educational Development; PTSD, posttraumatic stress disorder.

^a^ Data are presented as number (percentage) of participants unless otherwise indicated.

^b^ Individual classification based on self-report.

^c^ Other includes race not specified.

### Between-Group Comparison of the Transition Between Neutral and Negative States

**Figure 2.**
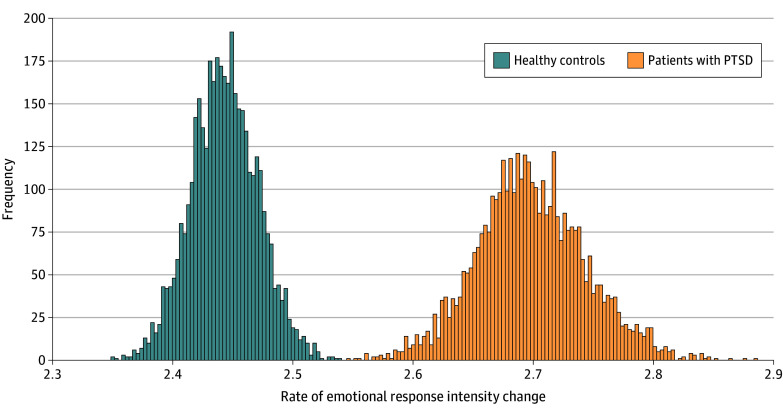
Transition From Neutral to Negative Valence in Patients With Probable Posttraumatic Stress Disorder (PTSD) Compared With Trauma-Exposed Controls The posterior distribution of the slope parameter of the 5-parameter logistic regression for the trauma-exposed control (TEC) and probable PTSD (pPTSD) groups. The higher slope in the pPTSD group indicates a quicker shift between neutral and negative states characteristic of PTSD.

The 5-PL model fits the data better in both the pPTSD and TEC groups (Table 2). A robust difference between the groups was found in the Hill slope parameter (Figure 2). The pPTSD group had higher slope rates (mean slope difference, −0.255; 89% HPD, −0.340 to −0.171), suggesting a steeper transition between neutral and negative valence. We found no evidence of a difference in any of the other parameters; see the eAppendix in Supplement 1 for more information and information regarding sensitivity analysis.

**Table 2.** Model Comparison Between a Linear and a 5-PL Regression

	Rank^a^	LOO^b^	p_loo^c^	d_loo^d^	Weight^e^	SE^f^
**TEC**						
5-PL	0	14 116.165	1619.063	0.000	0.895	168.598
Linear	1	12 894.716	1185.752	1221.449	0.105	152.060
**pPTSD**						
5-PL	0	5635.460	764.805	0.000	0.887	106.841
Linear	1	5040.644	582.490	594.815	0.113	98.508

Abbreviations: 5-PL, 5-parameter logistic; LOO, leave-one-out; pPTSD, probable posttraumatic stress disorder; TEC, trauma-exposed control.

^a^ The position of a model based on its LOO cross-validation score, with 0 being the best.

^b^ A higher LOO indicates better model fit because it reflects the model’s predictive accuracy.

^c^ Effective number of parameters. The p_loo represents the model complexity, with higher values indicating more complex models.

^d^ Difference in LOO between the model and the best model. The d_loo shows how much worse a model’s LOO score is compared with the best model’s LOO.

^e^ Model weights based on LOO scores. Weight indicates the relative importance or contribution of each model in model averaging.

^f^ The SE of the LOO estimate reflects the uncertainty in the LOO score, with larger values indicating more uncertainty.

### Association of Transitions Between Valence States With Emotional Numbing

**Figure 3.**
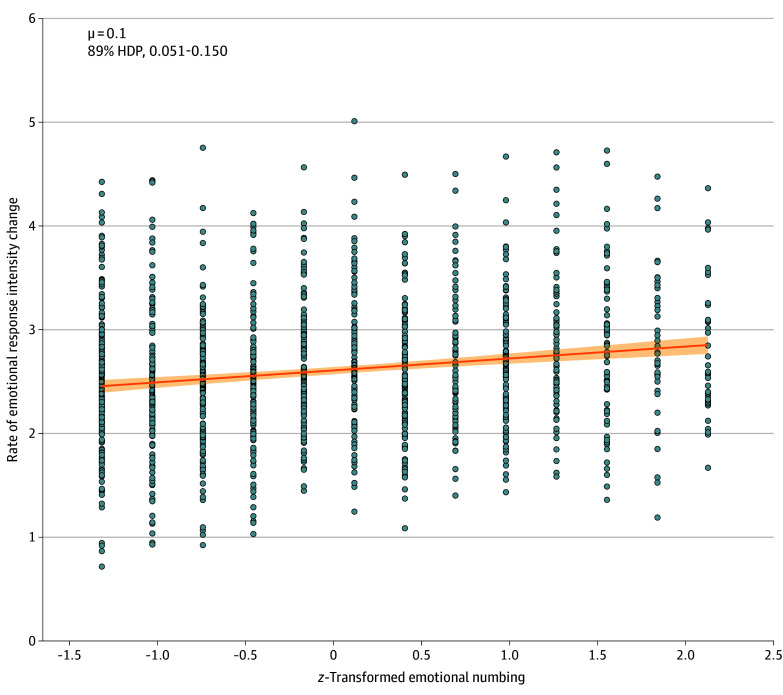
Association of the 5-Parameter Logistic Regression Slope With the Emotional Numbing Severity Score Associations are shown for the severity of emotional numbing, items 12 to 14 on the posttraumatic stress disorder checklist for *Diagnostic and Statistical Manual of Mental Disorders* (Fifth Edition) (*z* transformed), and the slope parameter from the 5-Parameter Logistic Regression. HDP indicates highest posterior density. Each individual point represents a participant’s data point. The orange line is the regression line, which reflects the mean association between emotional numbing and the rate of emotional response intensity change across all participants. The shading around the line represents the 95% credible interval for the regression slope.

Consistent with our hypothesis, we found a robust positive association between the regression slope (b) and the emotional numbing symptom severity in the entire sample (mean [SD] additive value, 0.100 [0.031]; 89% HPD, 0.051-0.150) (Figure 3). Because the groups differed in age, we tested additional models with (1) age and (2) interaction between age and emotional numbing terms. Age was *z*-transformed to improve convergence. The model comparison showed that the model with only emotional numbing better fit the data (see eTable 2 in Supplement 1 for model comparison and sensitivity analysis). Furthermore, an investigation of the model that included age and interaction terms suggested that emotional numbing substantially contributed to the regression slope (mean [SD] additive value, 0.108 [0.032]; 89% HPD, 0.056-0.159). In contrast, there was no compelling evidence of a robust contribution of age (mean [SD] additive value, 0.031 [0.033]; 89% HPD, −0.022 to 0.083) or the interaction term (mean [SD] additive value, −0.035 [0.033]; 89% HPD, −0.085 to 0.016).

## Discussion

This study aimed to investigate whether a more rapid transition between neutral and valence states can explain the hypo-hyper emotionality paradox in PTSD (Figure 1) and whether this transition is associated with emotional numbing symptoms. Using computational tools, we showed that, compared with trauma-exposed individuals without PTSD, those with pPTSD showed a steeper transition between neutral and negative valence states when rating the valence of images. As expected, this transition was associated with the severity of emotional numbing symptoms. These preliminary results provide valuable insights into the complex association between emotional processing and PTSD symptoms.

Previous studies have focused on the average rating of images in the same group. Images were grouped as negative trauma related compared with negative non–trauma related^7,36^ or based on the emotion they elicit.^37^ On the basis of this approach, the studies converged on the conclusion that only in trauma-related stimuli was there an increase in valence ratings. Our study partly supports this conclusion because we found no robust differences between groups when examining the lower (a) and upper (d) asymptote parameters, thus increasing the validity of our data set. Moreover, this finding corroborates leading theories that suggest that people diagnosed with PTSD can feel the entire spectrum of emotions^6^ and, most importantly, that when investigating the difference between people diagnosed with PTSD and healthy controls, we should focus on the transition between emotions and not whether they can feel or how much they feel.

Indeed, examining the transition between the states, we see an increase in the slope among individuals who based on self-reports met the criteria for PTSD compared with TECs, indicative of a more rapid shift. A gradual shift between states allows people to prepare themselves and use defensive mechanisms or emotion regulation. In contrast, a more rapid transition might leave people ill-equipped to handle the situation because it blocks the ability to exit the situation as it intensifies, leading to symptoms such as anger outbursts.^38^ The association between emotional numbing and the transition slope points to a response that is similar to that of pain. Where early response is inhibited, a high threshold leaves the individual vulnerable to more intense stimuli. The steeper transition slope between emotional states in PTSD and emotional numbing suggests a distinct pattern of emotional vulnerability. Furthermore, when drawing on the Research Domain Criteria framework,^39^ it becomes evident that PTSD shares processes with other psychological conditions. This finding suggests that the mechanisms underpinning PTSD, particularly those related to emotional numbing and irritability, are likely not exclusive to PTSD but are shared across various mental conditions characterized by numbing and irritability.^40,41^ This insight has implications for targeted therapeutic strategies, particularly in emotion regulation and mindfulness, to better manage this abrupt emotional transition and its associated symptoms. Therapists could assist patients in recognizing their states of emotional numbing. By fostering an understanding of these states, patients may learn to view their experiences from a new angle, preparing them to proactively disengage from potentially overwhelming situations before they exceed their threshold of tolerance.

Our findings support the hypothesis that engaging in the suppression of mild negative emotions (higher threshold) may contribute to the subsequent overexpression of emotions (lower tolerance). The opioid system may be at the core of this paradox. During periods of rest, people diagnosed with PTSD had lower^42^ or regular^43^ plasma-endorphin tone. However, after moderate stress (exercise), people diagnosed with PTSD had a robust increase in plasma-endorphin tone.^43^ Together with the higher binding potential of μ-opioid receptors in the amygdala,^44^ this increase can explain the amygdala’s lower response to mild pain in PTSD.^14^ Moreover, endorphin-mediated inhibition of the amygdala to lower valence images can potentially account for the sharper transition between neutral and negative states. Because the body’s defense mechanisms are already in action when low valence images are presented (ie, higher levels of endorphins), when higher valence images are shown, the amygdala receptors are saturated and thus cannot exert their calming effects, which results in the high-threshold, low-tolerance pattern of response.

### Limitations

This study has some limitations. First, although online studies enable access to populations that may not typically participate in laboratory-based research, there is a potential for data manipulation or lower data quality. To mitigate this limitation, we removed individuals who were deemed outliers and used attention checks. Second, there were age differences between the 2 groups. To address this difference, we ran an additional analysis to explore whether the age difference could explain the emotional numbing and slope association. We found that the model without age showed a better fit to the data (eAppendix in Supplement 1), and emotional numbing contribution remained robust even when age was introduced to the model. Third, we did not collect information on the index trauma, trauma load, childhood trauma, and time since trauma, limiting our ability to assess potential variations in the association between emotional numbing and the transition between valence states based on different aspects of the trauma. Fourth, in this proof-of-concept study, we used non–trauma-specific images. Nonetheless, the results demonstrate promising findings, and future research will explore whether incorporating trauma-specific stimuli yields a more robust association between emotional numbing and the transition between valence states.

## Conclusions

This study advances our understanding of emotional processing in PTSD by focusing on the transitional dynamics between neutral and negative emotional states, a facet often overlooked in traditional research. These transitional dynamics’ robust association with the severity of emotional numbing symptoms extends the prevailing understanding of emotional experiences in PTSD beyond intensity metrics. The steeper slope in emotional transitions holds notable clinical implications, directing attention toward developing targeted interventions that focus on emotion regulation or mindfulness strategies to modulate this pattern of high-threshold, low-tolerance responses.

## Notes

Supplement 1. **eTable 1.** List of Images and Average Rating Based on the NAPS **eFigure.** Effects of the 5-Parameter Logistic Regression **eTable 2.** Model Comparison **eAppendix.** Supplementary Sensitivity Analysis
eReferences

Supplement 2.
Data Sharing Statement
