# Supplementary material for: Emotional State Transitions in Trauma-Exposed Individuals With and Without Posttraumatic Stress Disorder
Source: JAMA Netw Open. 2024 Apr 16;7(4):e246813. doi: 10.1001/jamanetworkopen.2024.6813 (PMC11022112; doi:10.1001/jamanetworkopen.2024.6813)
Supplement: Supplement 1. — eTable 1. List of Images and Average Rating Based on the NAPS eFigure. Effects of the 5-Parameter Logistic Regression eTable 2. Model Comparison eAppendix. Supplementary Sensitivity Analysis eReferences [file jamanetwopen-e246813-s001.pdf]

Supplementary Online Content

Korem N, Duek O, Spiller T, Ben-Zion Z, Levy I, Harpaz-Rotem I. Emotional state transitions in trauma-exposed individuals with and without posttraumatic stress disorder. *JAMA Netw Open*. 2024;7(4):e246813. doi:10.1001/jamanetworkopen.2024.6813

eTable 1. List of Images and Average Rating Based on the NAPS
eFigure. Effects of the 5-Parameter Logistic Regression
eTable 2. Model Comparison
eAppendix. Supplementary Sensitivity Analysis
eReferences

This supplementary material has been provided by the authors to give readers additional information about their work.

© 2024 Korem N et al. *JAMA Network Open.*

eTable 1. List of images and average rating based on the NAPS[1]

Picture name	Valence rating	Picture name	Valence rating
People_238_h	1.33	Faces_296_h	3.64
People_198_h	1.58	People_005_h	3.74
Animals_074_h	1.70	People_210_h	3.84
People_226_h	1.81	Faces_280_h	3.94
Faces_159_h	1.93	Animals_052_v	4.04
People_205_v	2.09	Faces_037_h	4.14
People_128_h	2.23	Faces_036_h	4.23
Animals_071_h	2.35	People_014_h	4.33
Faces_293_h	2.44	People_076_h	4.43
Animals_027_h	2.55	Animals_142_h	4.53
Faces_153_v	2.65	Animals_069_h	4.65
Faces_170_h	2.74	Faces_171_v	4.75
Faces_147_v	2.84	Faces_167_v	4.84
Animals_032_h	2.96	Animals_047_h	4.94
People_023_h	3.06	Faces_312_h	5.04
People_012_h	3.16	Faces_078_h	5.63
Faces_011_h	3.25	Faces_203_h	6.11
People_071_h	3.35	Animals_149_v	7.00
People_070_v	3.45	People_169_h	7.58
Animals_050_h	3.55	Animals_117_h	7.09

© 2024 Korem N et al. *JAMA Network Open.*

eFigure. Effects of the 5-parameter logistic regression.

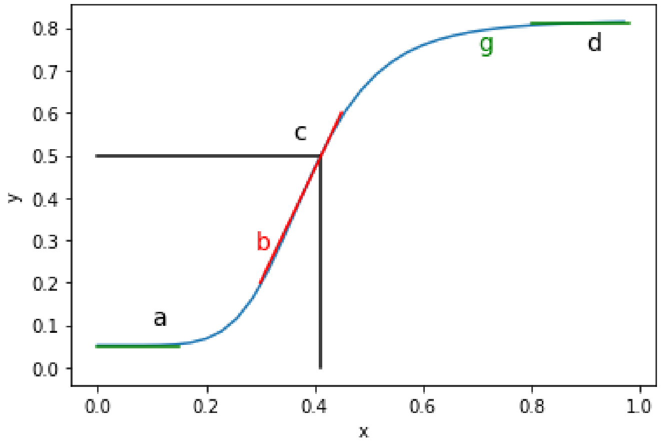

The 5-parameter logistic regression model (5PL) is designed to capture the complexity of data that exhibits an S-shaped curve with asymmetry. Each parameter within the model has a specific role in shaping the curve to fit the data accurately:

- **'a' (Minimum Asymptote):** This parameter represents the lowest point on the curve, which corresponds to the ratings of the most negative images in our context. It reflects the baseline level from which the curve starts.
- **'b' (Hill's Slope):** Named after Archibald Hill, this parameter determines the steepness or slope of the curve. A higher 'b' value indicates a steeper ascent, illustrating how quickly the response shifts from the minimum to the maximum asymptote as the stimulus changes.
- **'c' (Inflection Point):** This is the point along the x-axis where the curve changes direction most rapidly, marking the transition from the curve's rising phase to its slowing growth phase. In the context of image rating, 'c' represents where participants perceive images to transition from negative to more neutral valence, approximately at the midpoint of the curve where y is around (d-a)/2.
- **'d' (Maximum Asymptote):** Opposite to 'a', 'd' represents the curve's upper limit, reflecting the highest ratings given to images. For neutral images, 'd' signifies the plateau of the curve where increases in stimulus no longer significantly change the response.
- **'g' (Asymmetry Factor):** This parameter adjusts the symmetry of the curve around the inflection point 'c'. A 'g' value of 1 indicates a perfectly symmetrical curve. Values different from 1 introduce asymmetry, allowing the model to accommodate data where the response's increase and decrease do not mirror each other exactly around 'c'.

Together, these parameters enable the 5PL model to flexibly fit data with an S-shaped distribution, accommodating both symmetrical and asymmetrical patterns, and providing a nuanced understanding of how participants' emotional responses transition across a spectrum of stimuli.

© 2024 Korem N et al. *JAMA Network Open.*

eTable 2. Model comparison

	rank	loo	p_loo	d_loo	Weight	SE
**Emotional numbing**	0	19412.525	2544.024	0.000	1	196.891
**EN and Age**	1	19409.709	2547.311	2.815	0	196.882
**Interaction**	2	19405.891	2551.005	6.634	0	196.914
**Age**	3	19404.898	2551.401	7.626	9.77e-14	196.967

The ArviZ model comparison table provides a comprehensive overview of different statistical models based on several metrics, enabling the comparison of their fit and complexity.[2] Here's a brief explanation of the columns in the table:

- **Rank:** Orders models based on their performance, with the model ranked 1 considered the best according to the Leave-One-Out Cross-Validation (LOO) score.
- **LOO:** Leave-One-Out Cross-Validation score, a measure of model predictive accuracy. Higher LOO values indicate better model performance.
- **p_LOO:** Effective number of parameters, representing model complexity. Lower p_LOO values suggest a simpler model.
- **d_LOO:** Difference in LOO between each model and the best model. A d_LOO of 0 indicates the top model.
- **Weight:** Indicates the relative likelihood of each model being the best model among those compared, based on their LOO scores.
- **SE:** Standard Error of the LOO score, providing an estimate of the uncertainty associated with the LOO calculation.

In this context, the model focusing solely on emotional numbing emerged as the best fit due to having the highest LOO score, which signifies superior predictive accuracy. Simultaneously, this model exhibited the lowest p_LOO, indicating it has the lowest complexity among the models compared. The combination of high LOO and low p_LOO suggests that it effectively captures the data patterns without unnecessary complexity, making it the preferred choice. This balance of simplicity and accuracy underscores its suitability for understanding the dynamics of emotional numbing within the studied phenomenon.

© 2024 Korem N et al. *JAMA Network Open.*

eAppendix. Supplementary sensitivity analysis

We conducted extensive sensitivity analyses to ascertain the robustness of our model, specifically examining the differences between the TEC and pPTSD groups under various conditions.

Parameter Sensitivity:[3] Initially, we varied the prior parameters to evaluate the model's stability. For the 'a' parameter within the beta distribution, we explored combinations of [(4, 20), (2, 5), (5, 5)]. For the 'b' parameter, which defines the mean of the truncated normal distribution, we tested [1, 1.5, 2]. Similarly, for the 'c', 'd', and 'g' parameters—also means of truncated normal distributions—we examined [1, 2, 3], [1.5, 2, 2.5], and [3, 4, 5], respectively. The posterior distributions were inspected across these variations, demonstrating that our model maintained its robustness, thereby reinforcing our findings. Detailed results can be found in Notebook 6 at our GitHub repository.

Sample Size Sensitivity: Additionally, we assessed the model's performance across different sample sizes, starting from 40 and increasing in increments of 40. Our analysis indicated that for sample sizes greater than 120, the results consistently exhibited robustness, further validating the reliability of our study's conclusions.

These comprehensive sensitivity tests—spanning both parameter adjustments and sample size variations—substantially bolster the credibility of our model and the derived conclusions regarding the TEC and pPTSD groups.

© 2024 Korem N et al. *JAMA Network Open.*

eReferences

1. Marchewka A, Żurawski Ł, Jednoróg K, Grabowska A. The Nencki Affective Picture System (NAPS): Introduction to a novel, standardized, wide-range, high-quality, realistic picture database. *Behav Res Methods*. 2014;46(2):596-610. doi:10.3758/s13428-013-0379-1
2. Kumar R, Carroll C, Hartikainen A, Martín OA. ArviZ a unified library for exploratory analysis of Bayesian models in Python. Published online 2019.
3. Kruschke JK. Bayesian Analysis Reporting Guidelines. *Nat Hum Behav*. 2021;5(10):1282-1291. doi:10.1038/s41562-021-01177-7

© 2024 Korem N et al. *JAMA Network Open.*
