# Supplementary material for: Emotional State Transitions in Trauma-Exposed Individuals With and Without Posttraumatic Stress Disorder
Source: JAMA Netw Open. 2024 Apr 16;7(4):e246813. doi: 10.1001/jamanetworkopen.2024.6813 (PMC11022112; doi:10.1001/jamanetworkopen.2024.6813)
Supplement: Supplement 2. — Data Sharing Statement [file jamanetwopen-e246813-s002.pdf]

Data Sharing Statement

Korem. Emotional State Transitions in Trauma-Exposed Individuals With and Without Posttraumatic Stress Disorder. *JAMA Netw Open*. Published April 16, 2024. doi:10.1001/jamanetworkopen.2024.6813

Data
Data available: Yes
Data types: Deidentified participant data
How to access data: https://osf.io/gy25z/
When available: With publication

Supporting Documents
Document types: Statistical/analytic code
How to access documents: https://github.com/KoremNSN/EmotionalNumbing
When available: With publication

Additional Information
Who can access the data: everyone
Types of analyses: any purpose
Mechanisms of data availability: online available
